# *Chlamydia trachomatis* neither exerts deleterious effects on spermatozoa nor impairs male fertility

**DOI:** 10.1038/s41598-017-01262-w

**Published:** 2017-04-25

**Authors:** Jenniffer Puerta Suarez, Leonardo R. Sanchez, Florencia C. Salazar, Hector A. Saka, Rosa Molina, Andrea Tissera, Virginia E. Rivero, Walter D. Cardona Maya, Ruben D. Motrich

**Affiliations:** 1 Centro de Investigaciones en Bioquímica Clínica e Inmunología (CIBICI-CONICET), Departamento de Bioquímica Clínica. Facultad de Ciencias Químicas, Universidad Nacional de Córdoba, Haya de la Torre y Medina Allende, Córdoba, 5016 Argentina; 2 Grupo Reproducción, Facultad de Medicina, Universidad de Antioquia, Sede de Investigación Universitaria, SIU, Laboratorio 534, 1226 Medellín, Colombia; 3 Laboratorio de Andrología y Reproducción, LAR, Córdoba 5016 Argentina

## Abstract

*Chlamydia trachomatis* is the most prevalent sexually transmitted bacterial infection. However, whether *Chlamydia trachomatis* has a negative impact on sperm quality and male fertility is still controversial. Herein, we report the effects on sperm quality of the *in vitr*o exposure of spermatozoa to *Chlamydia trachomatis*, and also the effects of male genital infection on male fertility using an animal model. Human and mouse sperm were obtained from healthy donors and cauda epididimys from C57BL/6 mice, respectively. Highly motile human or mouse spermatozoa were *in vitro* exposed to *C. trachomatis* (serovar E or LGV) or *C. muridarum*, respectively. Then, sperm quality parameters were analyzed. Moreover, male fertility of *Chlamydia muridarum* infected male C57BL/6 mice was assessed. Human or murine sperm *in vitro* exposed to increasing bacterial concentrations or soluble factors from *C. trachomatis* or *C. muridarum*, respectively, did not show differences in sperm motility and viability, apoptosis, mitochondrial membrane potential, DNA fragmentation, ROS production and lipid peroxidation levels, when compared with control sperm (*p* > 0.05). Moreover, no differences in fertility parameters (potency, fecundity, fertility index, pre- and post-implantation loss) were observed between control and infected males. In conclusion, our results indicate that *Chlamydia* spp. neither directly exerts deleterious effects on spermatozoa nor impairs male fertility.

## Introduction

*Chlamydia trachomatis* (CT) is the most prevalent sexually transmitted bacterial pathogen in humans and the most common infection among sexually active young adults^1^. Yearly, approximately 100 million new cases of genital CT infections are diagnosed worldwide, although this is probably an underestimation^2^. Moreover, up to 90% infections in women and 50% in men are subclinical or asymptomatic^1^, favoring bacterial spread. The prevalence of chlamydial genital infections is similar in males and females; however, most current research in the field and screening strategies have been mainly focused on females^3^, neglecting the importance of CT infections of the male genital tract (MGT).

In the natural infection, male to female/male transmission is due to chlamydial elementary bodies (EBs) that are free in the seminal plasma or in infected epithelial cells from the MGT during sexual intercourse. Clinical manifestations in women include acute urethritis, bartholinitis, mucopurulent cervicitis, endometritis, salpingitis, pelvic inflammatory disease, perihepatitis, periappendicitis and reactive arthritis^1,4^. Untreated infections in women lead to severe reproductive complications including pelvic inflammatory disease, chronic pelvic pain, ectopic pregnancy, miscarriage and tubal infertility^4^. In men, CT causes urethritis, prostatitis, epididymitis and epididymis-orchitis^5,6^. Moreover, chronic chlamydial male genital infections have been suggested as possible causes of infertility in recent years^7,8^. However, whether CT infections of the MGT impair sperm quality and male fertility is a controversial issue^6,7^. A clear association of CT infections of the MGT and male infertility has not yet been proven^6–10^. Some *in vitro* studies have proposed that CT interacts with sperm cells, affecting their function and inducing apoptosis^11–15^. However, these findings had not been reproduced in *in vivo* studies^16^. Besides, some evidence published over two decades ago showed a possible direct interaction and internalization of CT into sperm cells^17,18^, with significant implications for assisted reproduction technology. To date, and to the best of our knowledge, similar results have not been reported. In summary, the limited available data is conflicting and compelling evidence establishing a clear relationship between CT infection and sperm/semen quality is currently lacking.

Herein, we report the effects on sperm quality of the *in vitr*o exposure of human and murine spermatozoa to CT and *Chlamydia muridarum*, respectively. Moreover, and using a mouse model of genital tract chlamydial infection, we assessed the effects of *C. muridarum* infection of the MGT on male fertility. Finally, we also evaluated if *Chlamydia* spp. attach to spermatozoa using *in vitro* and *in vivo* approaches.

## Results

### *Chlamydia* spp. does not impair sperm motility and viability

**Figure 1:**
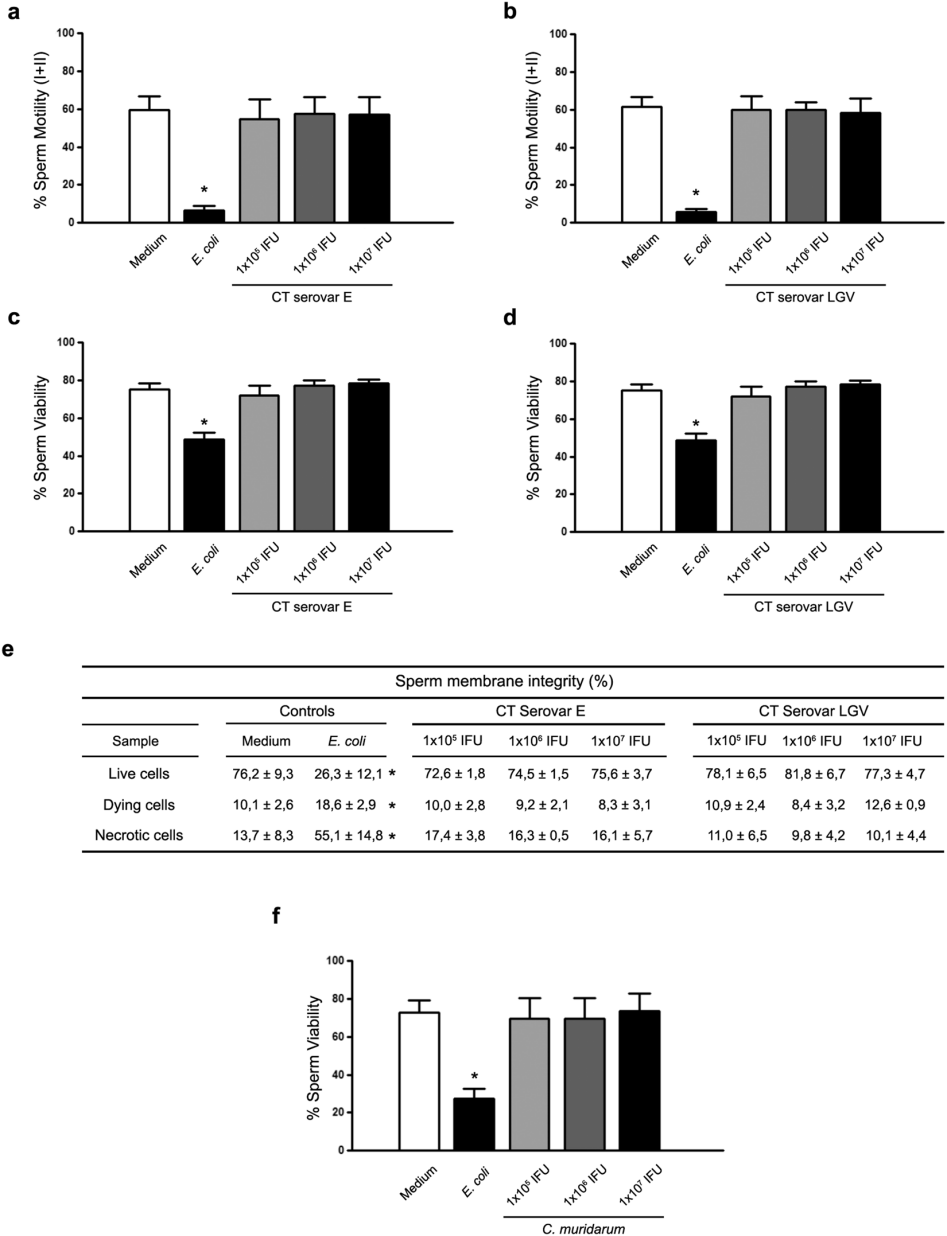
Effects of *Chlamydia* spp. on sperm motility and viability. Human sperm motility (%) after 6 h of *in vitro* incubation without bacteria (Control), or with increasing concentrations of EBs of CT serovar E (**a**) or serovar LGV (**b**) per million spermatozoa. Human sperm viability (%) after 6 h of *in vitro* incubation without bacteria (Control), or with increasing concentrations of EBs of CT serovar E (**c**) or serovar LGV (**d**) per million spermatozoa. (**e**) Sperm membrane integrity (%) in human spermatozoa after *in vitro* incubation without bacteria (Control), or with increasing concentrations of EBs of CT serovar E or serovar LGV per million spermatozoa. (**f**) Murine sperm viability (%) after 30 min of *in vitro* incubation without bacteria (Control) or with increasing concentrations of EBs of *C. muridarum* per million spermatozoa. As positive controls, sperm fractions were incubated with uropathogenic *E. coli* (1 × 10^6^ CFU/mL). Data are shown as mean ± SD. Fractions of human (n = 54) and mouse (n = 24) sperm samples were tested separately and maintained at 37 °C throughout all procedures. Statistical analysis was performed using one-way ANOVA with Bonferroni post hoc test analysis and no significant differences were found in any condition (p < 0.05).

Figure 1a–e shows the effects on sperm motility and viability of the co-incubation of human sperm with three different concentrations of CT serovar E or LGV during 6 h. As shown, similar levels of sperm motility were observed in human sperm incubated with either CT serovar E or LGV as those observed in control sperm (sperm incubated with medium alone) (Fig. 1a,b). On the contrary, as previously reported^19^, the exposure of human sperm to 1 × 10^6^ CFU/ml of viable *Escherichia coli* significantly decreased sperm motility (Fig. 1a,b). In addition, similar results were observed when analyzing sperm viability (Fig. 1c,d). Accordingly, sperm plasma membrane integrity evaluation, as an additional measure of sperm viability, showed no significant differences between control spermatozoa (incubated with medium alone) and spermatozoa co-incubated with CT, either serovar E or LGV (Fig. 1e). Furthermore, similar results were observed when assaying highly motile fractions of human sperm samples *in vitro* exposed to CT, either serovar E or LGV, during 24 h (Supplementary Fig. 1). Since soluble factors of other bacteria such as *E. coli* have been shown to alter sperm^19^, the effects of soluble factors of CT on sperm motility and viability were also analyzed. Fractions of highly motile human spermatozoa were co-incubated with supernatants obtained from HeLa cell cultures after 48 h of infection with CT serovar E or LGV at three different concentrations. Mean values of motility or viability from spermatozoa exposed to soluble factors from CT, either serovar E or LGV, showed no significant differences when compared with values from spermatozoa co-incubated with supernatants from uninfected cultures (Medium) (Supplementary Fig. 2).

In agreement with these results, *C. muridarum* did not exert deleterious effects on murine sperm viability (Fig. 1f).

### *Chlamydia* spp. does not induce sperm apoptosis

*In vitro* exposure of spermatozoa to CT did not induce sperm apoptosis since similar levels of early (A+/PI−) or late (A+/IP+) apoptosis were observed in human spermatozoa incubated with either CT serovar E or LGV, at any bacterial concentration assessed, when compared with control spermatozoa (Table 1). Moreover, levels of early or late apoptosis in spermatozoa incubated with cLPS were similar to those observed in control spermatozoa (Table 1). However, significantly elevated levels of early apoptosis were detected in human spermatozoa incubated with *E. coli* or with LPS purified from *E. coli* (*E. coli* LPS) (Table 1), as previously reported^19^. Furthermore, similar results were observed when analyzing murine spermatozoa incubated with *C. muridarum*, cLPS, *E. coli* LPS, or *E. coli* (Table 1).

**Table 1:** *Chlamydia* spp. and sperm apoptosis.

Sperm Apoptosis^a^						
Experimental condition			Live (A−/PI−)	Live, early apoptotic (A+/PI−)	Dead, late apoptotic/early necrotic (A+/PI+)	Dead, Late necrotic (A−/PI+)
Human sperm	Vehicle		76.9 ± 2.5	20.3 ± 3.3	2.1 ± 0.7	0.7 ± 0.4
	*Chlamydia trachomatis* Serovar E	1 × 10^5^ EBs	71.7 ± 3.3	21.2 ± 0.7	3.8 ± 1.6	3.3 ± 1.6
		1 × 10^6^ EBs	72.0 ± 3.0	23.7 ± 4.2	3.2 ± 0.7	1.1 ± 0.5
		1 × 10^7^ EBs	68.7 ± 2.8	19.7 ± 7.0	5.2 ± 1.4	6.4 ± 5.3
	*Chlamydia trachomatis* Serovar LGV	1 × 10^5^ EBs	69.0 ± 2.7	25.6 ± 1.5	3.8 ± 1.6	1.6 ± 1.1
		1 × 10^6^ EBs	74.2 ± 4.0	20.6 ± 4.2	4.3 ± 0.5	0.9 ± 0.4
		1 × 10^7^ EBs	79.7 ± 2.3	14.7 ± 2.8	4.2 ± 0.3	1.4 ± 0.4
	Chlamydial LPS		72.3 ± 0.4	22.0 ± 2.6	4.4 ± 1.6	1.3 ± 0.7
	*E. coli* LPS		36.8 ± 5.7*	37.8 ± 7.2*	17.6 ± 3.6*	7.8 ± 13.5*
	*E. coli*		37.1 ± 5.9*	28.3 ± 6.9*	24.9 ± 6.4*	9.7 ± 2.9*
Murine sperm	Vehicle		59.0 ± 5.5	12.9 ± 6.2	27.5 ± 3.3	0.6 ± 0.4
	*Chlamydia muridarum*	1 × 10^5^ EBs	54.7 ± 7.6	8.2 ± 4.2	35.9 ± 4.4	1.2 ± 0.6
		1 × 10^6^ EBs	53.0 ± 3.8	15.3 ± 6.3	31.1 ± 3.1	0.6 ± 0.1
		1 × 10^7^ EBs	55.4 ± 9.2	14.6 ± 5.5	29.2 ± 7.5	0.8 ± 0.7
	Chlamydial LPS		58.8 ± 7.9	18.9 ± 3.5	22.0 ± 6.1	0.3 ± 0.1
	*E. coli* LPS		12.9 ± 3.4*	72.8 ± 8.8*	13.9 ± 5.3*	0.4 ± 0.1
	*E. coli*		18.7 ± 4.6*	26.5 ± 16.5	51.4 ± 13.6*	3.4 ± 2.6*

^a^Levels (%) of apoptosis/necrosis in human and murine sperm after 6 h or 30 min, respectively, of *in vitro* incubation with increasing concentrations of *Chlamydia* spp. EBs, or with cLPS, *E. coli* LPS, or 1 × 10^6^ CFU of *E. coli*/million sperm. Data are shown as mean ± SD. Human (n = 54) and mouse (n = 24) sperm samples were tested separately and maintained at 37 °C throughout all procedures. Statistical analysis was performed using one-way ANOVA with Bonferroni post hoc test analysis. *p < 0.05.

As shown in Table 2, no significant differences were observed in sperm mitochondrial functionality (ΔΨm) or DNA fragmentation levels in human spermatozoa co-incubated with CT, either serovar E or LGV, when compared with control spermatozoa (vehicle). Similar results were observed when analyzing the effects of soluble factors from CT serovar E or LGV (Supplementary Table 1). Altogether, these data indicate that *Chlamydia* spp. do not exert toxic effects on spermatozoa under the experimental conditions assayed.

**Table 2:** Effects of CT on the mitochondrial physiology and DNA integrity of human sperm.

Sperm mitochondrial membrane potential, ΔΨm (%)^a^										
CT	Serovar E					Serovar LGV				
Sample	Vehicle	Control+	1 × 10^5^ EBs	1 × 10^6^ EBs	1 × 10^7^ EBs	Vehicle	Control+	1 × 10^5^ EBs	1 × 10^6^ EBs	1 × 10^7^ EBs
High ΔΨm	54.2 ± 3.8	45.2 ± 2.9*	55.0 ± 3.9	55.7 ± 8.4	51.1 ± 6.9	63.3 ± 6.2	48.3 ± 6.5*	67.5 ± 15.5	59.7 ± 5.4	58.3 ± 6.8
Low ΔΨm	20.1 ± 6.5	7.7 ± 2.2*	21.1 ± 4.4	19.1 ± 8.3	23.0 ± 7.8	16.2 ± 4.5	6.5 ± 2.7*	12.3 ± 7.0	20.0 ± 8.3	20.0 ± 7.3
**Sperm DNA fragmentation** ^**b**^										
DNA fragmentation index	4.0 ± 1.0	15.6 ± 0.7*	4.2 ± 0.6	4.3 ± 2.2	3.5 ± 2.6	3.1 ± 2.6	17.1 ± 1.1*	4.1 ± 1.3	5.3 ± 3.6	2.9 ± 1.3

Percentages of human sperm exhibiting high and low mitochondrial membrane potential (ΔΨm)^a^, and evaluation of DNA fragmentation^b^, in human sperm samples after 6 h of *in vitro* incubation without bacteria (Vehicle), or with increasing concentrations of EBs of CT serovar E or serovar LGV. Data are shown as mean ± SD, n = 36. Statistical analysis was performed using one-way ANOVA with Bonferroni post hoc test analysis. *p < 0.05.

### CT neither induces sperm ROS production nor membrane lipid peroxidation

**Figure 2:**
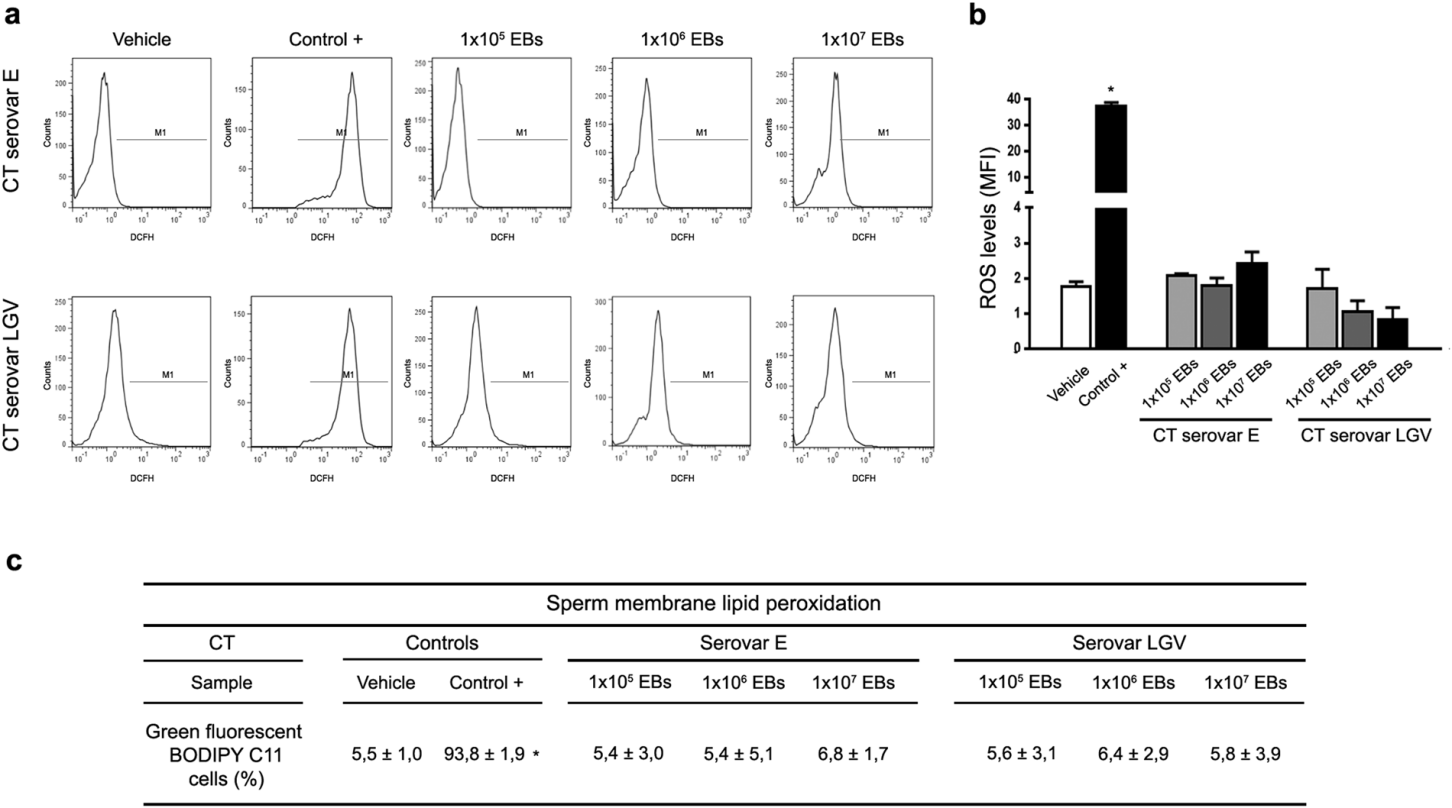
ROS production and lipid peroxidation in human spermatozoa exposed to CT. Analysis of ROS production by human spermatozoa after 6 h of *in vitro* incubation without bacteria (Vehicle), 100 nM PMA (positive control) or with increasing concentrations of EBs of CT serovar E or serovar LGV per million spermatozoa. ROS production was assessed by flow cytometry using the probe DCFH-DA that fluoresces when oxidized to DCFH. (**a**) Histograms show sperm with ROS production (M1). (**b**) ROS production levels (MFI) are shown in bars. (**c**) Human sperm membrane lipid peroxidation (%) after 6 h of *in vitro* incubation without bacteria (Vehicle), 100 mM TBHP (positive control) or with increasing concentrations of EBs of CT serovar E or serovar LGV per million spermatozoa. Lipid peroxidation was analyzed using BODIPY C11. Data are shown as mean ± SD. Fractions of sperm samples (n = 36) were tested separately. Statistical analysis was performed using one-way ANOVA with Bonferroni post hoc test analysis. *p < 0.05.

Since it has been reported that bacteria such as *E. coli* induce sperm ROS production and membrane lipid peroxidation^20^, ROS production and lipid peroxidation were analyzed in human spermatozoa co-incubated with different concentrations of CT, serovar E or LGV, or their soluble factors. No significant differences were observed in either ROS production (Fig. 2a,b) or membrane lipid peroxidation (Fig. 2c) levels in spermatozoa co-incubated with CT serovar E or LGV when compared with their respective control counterparts (vehicle), or when assaying soluble factors of CT serovar E or LGV (Supplementary Fig. 3). Hence, CT does not induce either sperm oxidative stress or membrane lipid peroxidation.

### *C. muridarum* infection of the male genital tract does not impair male fertility

Considering results shown above, we then aimed to test the effects of chlamydial infection of the MGT on male fertility *in vivo* using an animal model in mice. In this regard, the development and study of murine models of *C. muridarum* infection of the MGT have been pioneered by our group^6,21–23^. These models have shown that inoculated rats and mice develop an ascending chlamydial infection that is detectable in semen and male genital tract tissues, showing special tropism for the prostate and persisting even until 80 dpi^21–23^. As previously described^23^, C57BL/6 male mice were inoculated in the meatus urethra with *C. muridarum* or vehicle, and mating experiments were performed to analyze different fertility parameters. Once mating experiments were performed, sham infected and infected male mice were euthanized to confirm the presence of infection by screening *C. muridarum* DNA in prostate tissue samples. *C. muridarum* was detected in 100% of prostate tissue samples from infected animals after 20 days of inoculation (Table 3). Negative results were obtained in prostate tissue samples from sham infected mice.

**Table 3:** Effects of *C. muridarum* infection of the male genital tract on male fertility.

Fertility parameters		
Parameter (mean ± SD)	Experimental groups	
	Control	Infected
*C. muridarum* infected males	0/6 (0%)	6/6 (100%)
% Potent	79.3	81.3
% Fecund	100	100
Fertility index	0.92 ± 0.06	0.93 ± 0.11
% embryo loss		
Before implantation	8.75 ± 6.52	7.87 ± 13.53
After implantation	14.38 ± 12.06	9.61 ± 12.20

Evaluation of fertility parameters of control and *C. muridarum* infected male mice. Six-to-eight week old male C57BL/6 mice were inoculated in the meathus urethra with 20 μL of vehicle (SPG, Control group, n = 6) or with a suspensión containing 1 × 10^8^ EBs of *C. muridarum* (Infected group, n = 6). After 15 days of inoculation, males were mated with sexually mature female C57BL/6 mice to evaluate fertility parameters. Every male was mated with 3 females (a total of 36 females, n = 18 females per experimental group). Data are shown as mean ± SD. Statistical analysis was performed using two-way ANOVA with Bonferroni post hoc test analysis (*p* > *0.05*).

Mating experiments with sexually mature female C57BL/6 mice revealed no significant differences in the ability of sham infected and infected male mice to mate (potency) (Table 3). Moreover, no significant differences were found in fecundity potential between the sham infected and infected groups. When the fertility index was analyzed, no significant difference was found between infected and control males (Table 3). In addition, females mated with infected males had similar pre-implantation and post-implantation embryo loss compared to the sham infected group (Table 3). Overall, these results indicate that *C. muridarum* infection of the MGT does not compromise male fertility.

### *Chlamydia* spp. do not attach to spermatozoa

Some controversial evidence published more than two decades ago suggested that CT tightly interacts with and/or internalizes into spermatozoa^17,18^, having significant implications for assisted reproduction technology. Taking this into consideration, we finally tested whether *Chlamydia* spp. is able to attach to spermatozoa by conducting different experimental approaches.

**Figure 3:**
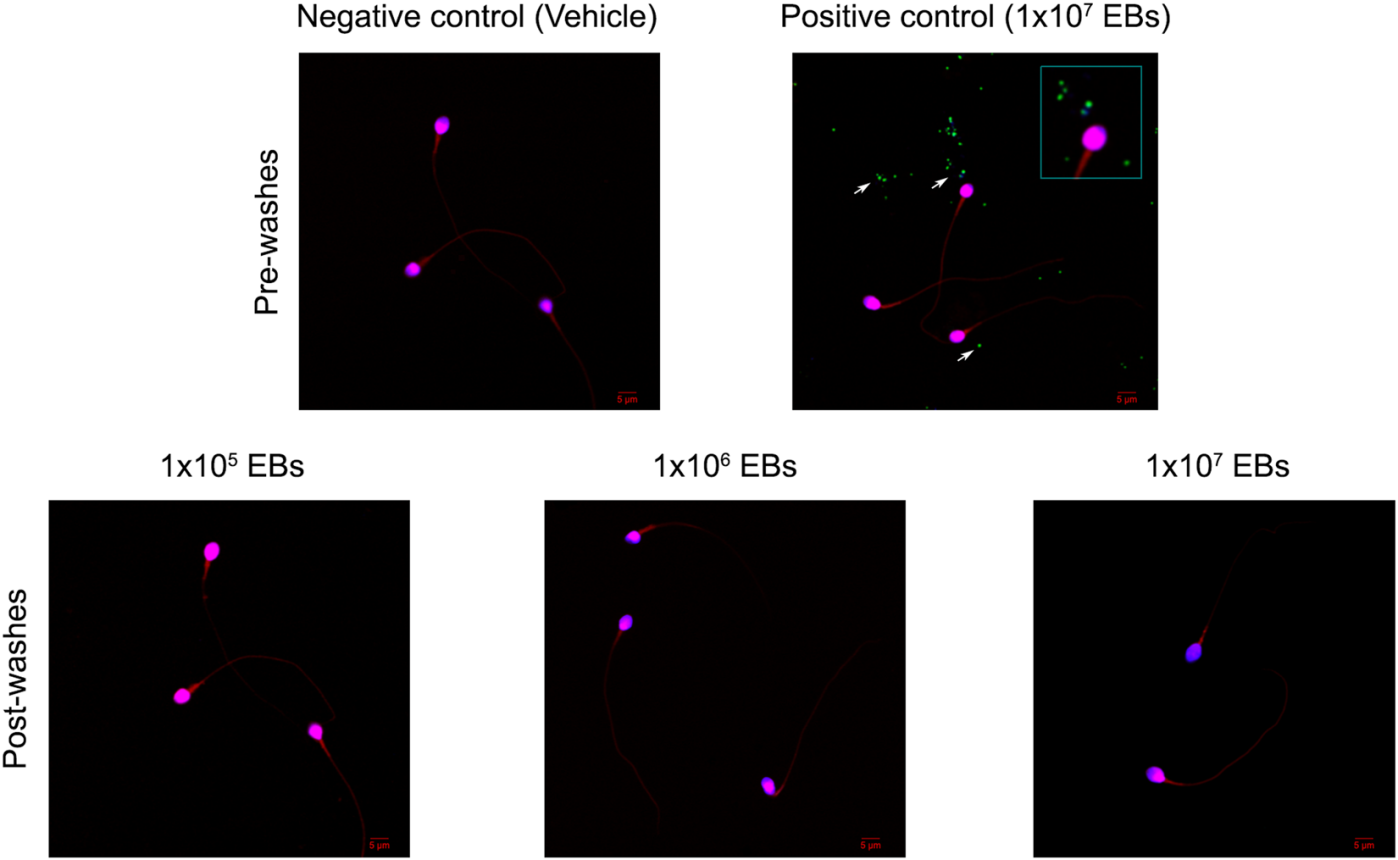
CT does not attach to spermatozoa *in vitro*. Fractions of highly motile human sperm samples containing 1 × 10^6^ sperm/ml were *in vitro* incubated with 3% BSA supplemented BWW medium alone or with 1 × 10^5^, 1 × 10^6^ and 1 × 10^7^ EBs/mL of GFP-CT (green fluorescent) during 6 h at 37 °C. Sperm suspensions were then subjected to five consecutive washes and centrifuged at 300 × *g* for 5 min to remove free bacteria (Post-washes). Sperm suspensions were smeared onto glass slides, stained with DAPI (stain for DNA) and counterstained with 0.01% Evans blue and analyzed by confocal microscopy. Negative control corresponds to fractions of sperm samples incubated with supplemented BWW medium alone [Negative control (Vehicle), Pre-washes]. Positive control corresponds to fractions of sperm samples incubated with 1 × 10^7^ EBs of GFP-CT without subsequent washings [Positive control (1 × 10^7^ EBs), Pre-washes]. Green fluorescent EBs of GFP-CT are observed as green/blue co-localizing dots (marked with arrows). Results shown are from one representative experiment out of three performed (using fractions of n = 10 different sperm samples) with essentially the same results. Images were captured using an Olympus FV1200 laser scanning confocal microscope with an objective PLAPON 60X (1.42 NA). Fluorophore signals were acquired in sequential mode.

First, fractions of human spermatozoa were incubated with different concentrations of green fluorescent EBs of CT (GFP-CT). Then, sperm suspensions were subjected to five consecutive washes with BSA-supplemented BWW medium to remove free bacteria, and smeared on slides that were analyzed by fluorescent confocal microscopy. As observed in Fig. 3, abundant EBs of GFP-CT (observed as green/blue co-localizing dots) were observed in fractions of sperm samples incubated with 1 × 10^7^ EBs/mL before washes (positive control, 1 × 10^7^ EBs pre-washes). Noteworthy, EBs of CT were observed free and detached from or not interacting with spermatozoa. Moreover, washing sperm suspensions with supplemented medium after incubation removed bacteria in every rate of bacterial concentration assayed indicating that CT does not attach to human spermatozoa *in vitro* [Fig. 3: compare 1 × 10^5^, 1 × 10^6^ or 1 × 10^7^ EBs post-washes with the negative control (Vehicle), pre-washes].

**Figure 4:**
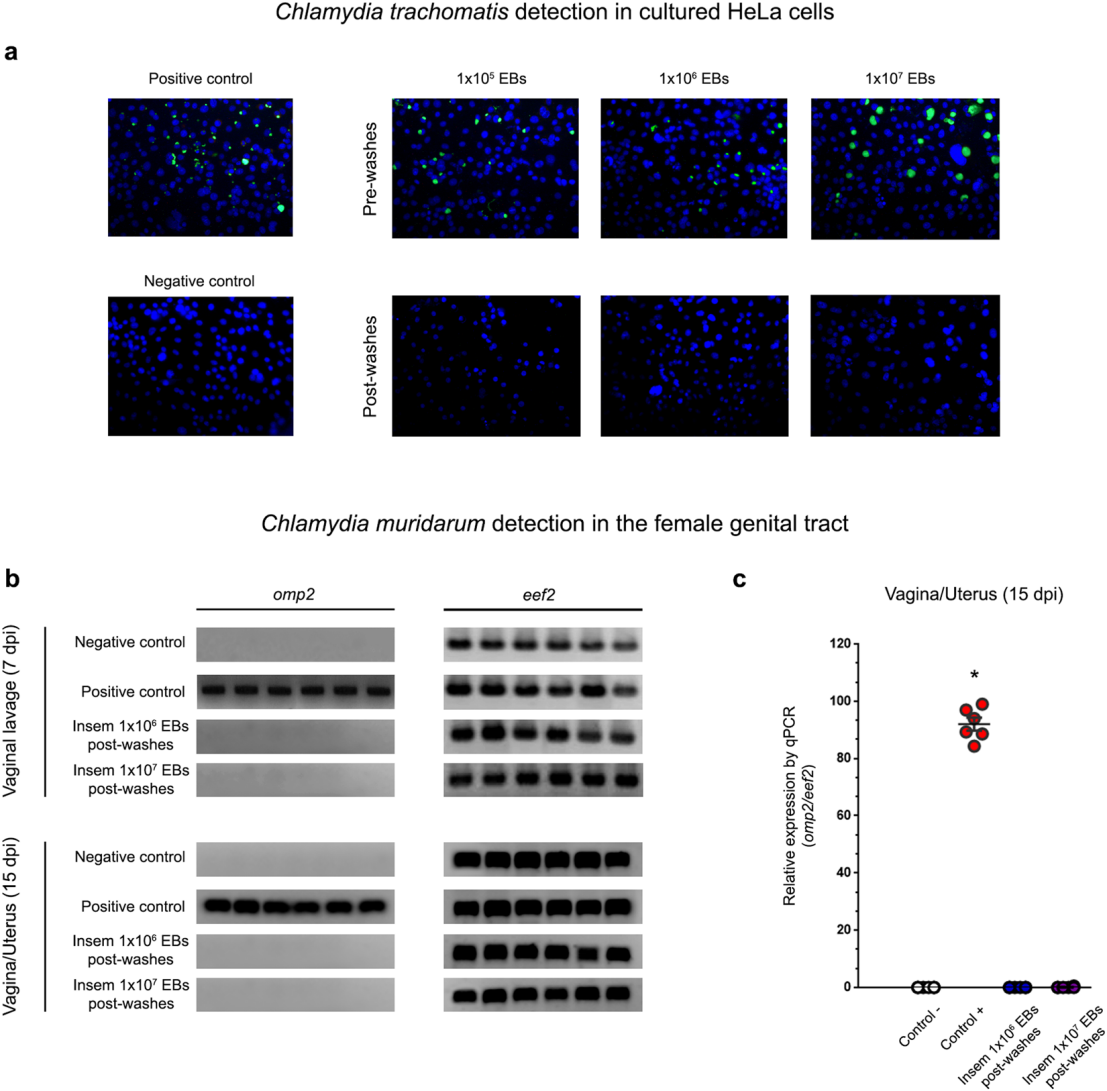
Spermatozoa do not carry attached EBs of *Chlamydia* spp. (**a**) *In vitro* cultured confluent HeLa cells were inoculated with human spermatozoa *in vitro* pre-incubated with different concentration rates (1 × 10^5^, 1 × 10^6^ or 1 × 10^7^ EBs/million sperm) of CT serovar E, immediately after incubation without removing bacteria (Pre-washes), or after five consecutive washes post-incubation (Post-washes) in order to remove free bacteria. Some cultured cells were directly inoculated with 1 × 10^6^ EBs of CT serovar E/mL (positive control) or with supplemented BWW medium alone (negative control). After 48 h of culture, infection of cultures was assessed by the detection of chlamydial inclusion bodies by direct immunofluorescence using a FITC-labeled anti-cLPS monoclonal antibody. Representative microphotographs of 1 out of 4 independent experiments performed with essentially the same results. (**b** and **c**) *C. muridarum* detection (*omp2* gene) by conventional (**b**) or real time PCR (qPCR) (**c**) in vaginal lavages (at 7 days post insemination) and female genital tract tissue samples (at 15 days post insemination) from female C57BL/6 mice that were intravaginally inseminated with murine sperm *in vitro* pre-incubated with *C. muridarum* in capacitating conditions and then washed 5 times to remove free bacteria. Four experimental groups were included: the sham infected group (n = 6) inseminated with 30 µl of a solution containing 1 × 10^6^ sperm that were pre-incubated with vehicle alone (BWW buffer); the positive control group (n = 6) inseminated with 30 µl of a solution containing 1 × 10^6^ sperm that were pre-incubated with 1 × 10^7^ EBs of *C. muridarum* without subsequent washings; one group (n = 6) inseminated with 30 µl of a solution containing 1 × 10^6^ sperm that were pre-incubated with 1 × 10^6^ EBs of *C. muridarum* and then washed 5 times; and one group (n = 6) inseminated with 30 µl of a solution containing 1 × 10^6^ sperm that were pre-incubated with 1 × 10^7^ EBs of *C. muridarum* and then washed 5 times. The expression of the housekeeping gene *eef2* was assayed. Representative data from 1 out of 3 independent experiments performed with essentially the same results. Data are shown as mean ± SD. Statistical analysis was performed using two-way ANOVA with Bonferroni post hoc test analysis. *p < 0.01.

Second, 1 × 10^6^ human spermatozoa were incubated with 1 × 10^5^, 1 × 10^6^ or 1 × 10^7^ EBs of CT serovar E. Then, sperm suspensions were subjected to five consecutive washes with BSA-supplemented BWW medium to remove free bacteria, and finally inoculated onto *in vitro* cultured HeLa cell monolayers. After 48 h, the presence of infection was investigated by performing immunofluorescent detection of chlamydial inclusions. Figure 4a shows that inoculation of cultures with either 1 × 10^6^ EBs of CT (positive control) or sperm incubated with three different concentrations of CT without posterior washes to remove free bacteria, resulted in the expected infection of HeLa cells. However, no infection was observed when inoculating cultures with spermatozoa pre-incubated with 1 × 10^5^, 1 × 10^6^ or 1 × 10^7^ EBs/million sperm and then washed to remove free bacteria (Fig. 4a). As expected, no infection was detected in cultures inoculated with control sperm (sperm incubated with medium alone, negative control). Thus, the fact that washing sperm suspensions (previously co-incubated with CT) prevented the posterior infection of inoculated HeLa cell cultures strongly suggests that CT does not attach to spermatozoa *in vitro*.

Lastly, an *in vivo* approach consisting in vaginal insemination of female C57BL/6 mice with mouse sperm *in vitro* pre-incubated with *C. muridarum* was conducted. *C. muridarum* infection was determined by assaying the expression of the chlamydial *omp2* gene by traditional PCR and quantitative PCR (qPCR) in vaginal lavages at 7 days post insemination (dpi) (Fig. 4b), and in vaginal and uterine tissue at 15 dpi (Fig. 4b,c). As expected, *C. muridarum* was detected in either vaginal lavages (7 dpi, Fig. 4b) or in vaginal/uterine tissue samples (15 dpi, Fig. 4b,c) from females inseminated with spermatozoa pre-incubated with 1 × 10^6^ *C. muridarum* EBs/million spermatozoa without further washes (Positive control). Conversely, no infection was detected in either vaginal lavages (7 dpi) (Fig. 4b) or in genital tract tissue samples (15 dpi) (Fig. 4b,c) from females inseminated with sperm suspensions pre-incubated with 1 × 10^6^ or 1 × 10^7^ EBs of *C. muridarum* EBs/million sperm that were then washed 5 times to remove free bacteria. Taken together, these results mirrored those from the *in vitro* experiments shown above, indicating that *Chlamydia* spp. do not attach to spermatozoa *in vitro*, which have significant implications for assisted reproduction technology.

## Discussion

Herein, using different experimental *in vitro* approaches and an animal model of infection, we provide evidence indicating that *Chlamydia* spp. neither significantly exerts deleterious effects on spermatozoa nor impairs male fertility *in vivo*. Our results show that CT serovars E and LGV do not impair human sperm motility and viability *in vitro*. Moreover, CT or its soluble factors including cLPS do not induce sperm apoptosis, mitochondrial dysfunction and DNA fragmentation. Also, no significant levels of either ROS production or membrane lipid peroxidation were detected in human sperm incubated with CT serovar E or LGV. Similar results were obtained when assaying murine sperm *in vitro* exposed to *C. muridarum*. Finally, and using a mouse model of infection^21–23^, no differences in the male fertility parameters assayed (potency, fecundity, fertility index, pre- and post-implantation embryo loss) were observed between control and *C. muridarum* infected males.

It has been estimated that 15% of male infertility is related to genital tract infection and inflammation^24^. Sexually transmitted infections account for 340 million new cases occurring each year worldwide^25^. Among them, the most prevalent bacterial infections are those caused by CT^9^. The impact of CT infection on female fertility has already been established^4,9^. While it is clear that the inflammation caused during chlamydial infection has detrimental effects on the female reproductive tract itself, it largely remains controversial if chlamydial infection has a significant effect on sperm quality and subsequently on male fertility^6–8^. In this regard, CT could directly damage the sperm by direct contact or its soluble products, or indirectly through the induction of tissue inflammation and the release of toxic inflammatory mediators such as ROS and cytokines. Thus, additional studies in patients and new studies research conducting *in vitro* and *in vivo* approaches are needed to improve the current understanding of this topic.

On one hand, some epidemiological reports indicate that CT infection of the MGT is associated with a decrease in sperm concentration, viability, motility and normal morphology^26,27^. Conversely, most other studies have revealed no significant association between CT infection of the MGT and altered sperm quality^28–33^. Interestingly, it has been shown that men co-infected with CT and *Mycoplasma* spp. had increased levels of sperm with fragmented DNA^34^. Nonetheless, these effects are more likely due to *Mycoplasma* spp. infection rather than CT infection^8,25^. Moreover, alterations in semen quality have been reported in prostatitis patients co-infected with CT and human papillomavirus (HPV) but not in patients only infected with CT^35^. Remarkably, the co-infection with other genital pathogens such as HPV, *Ureaplasma* spp., or *Mycoplasma* spp. rather than the sole CT infection appears to have deleterious effects in semen quality.

On the other hand, evidence from animal models of CT infection of the MGT is scarce. Recently, Sobinoff *et al*. showed that intrapenile inoculation with *C. muridarum* in mice caused an ascending infection and, once in testes, it damaged male germ cells and Sertoli cells 4–8 weeks post infection^36^. However, the semen quality of these infected animals was not assessed. This would have been an interesting aspect to be addressed in order to ascertain if the infection had an impact on semen quality. Besides, intrapenile inoculation of bacteria could be forcing infection rather than reflecting the course of natural infections. In experimental models of MGT infection, it is important to induce the infection through the natural route to obtain results comparable to natural infections. In fact, and mirroring natural infections, the inoculation of *C. muridarum* in the meatus urethra of male rats and mice causes an ascending infection of the MGT^21–23^. Semen analysis of infected animals showed that, either acute or chronic CT infection of the MGT, have no detrimental consequences in sperm quality, either from semen or epididymis^22^. Moreover, these animals showed to have their male fertility potential unimpaired when compared to control non-infected males^22^.

Some *in vitro* studies have proposed that CT interacts with sperm cells, affecting their function and inducing apoptosis, effects that were reported to be more pronounced for serovar E than for LGV^12–15^. Hosseinzadeh *et al*. indicated that these effects were induced by live bacteria but not by bacterial soluble products or membrane components, since the effects were not observed when co-incubating spermatozoa with heat-killed CT^12^. However, in a posterior work, these authors showed that LPS from both CT serovars, E and LGV, provoked a marked reduction in sperm motility similar to that observed in response to EBs from CT serovar E^13^. Moreover, they also proposed that chlamydial LPS, through its binding to CD14 on the sperm surface, could trigger ROS production and caspase-mediated apoptosis^13,14^, clearly contradicting their previous findings. Furthermore, these authors failed to confirm their findings in *in vivo* studies^16^ and recently reported no significant differences in sperm quality and inflammatory cytokines levels in semen from control and CT-infected male individuals^28^. Also, they reported that current or past CT infections in male partners of infertile couples have no influence on the chance of pregnancy or pregnancy outcome^37^.

Noteworthy, most conflicting data on CT and sperm quality come from the available *in vitro* studies. Therefore, additional and compelling evidence is needed to clearly establish whether CT infection alters sperm quality and male fertility. Herein, we provide new evidence indicating that the direct *in vitro* exposure of spermatozoa to CT neither impairs sperm motility and viability nor induces sperm apoptosis. In addition, CT or its soluble factors including cLPS do not induce sperm apoptosis, mitochondrial dysfunction or DNA fragmentation. Most of these findings are supported by similar results observed when analyzing murine spermatozoa *in vitro* exposed to *C. muridarum*, the particular species of *Chlamydia* that naturally infect rodents^6^. Interestingly, our results are in contrast to some previously reported data^12–15^. Differences in experimental conditions and samples used may account, at least in part, for the discrepancies observed. Nevertheless, our results are strengthened by having obtained consistent results evaluating different conventional, non-conventional and functional sperm parameters, broadly different doses of different species and biovars of *Chlamydia* spp., incubation times, and sperm from different species. Our results also show that CT neither induces sperm ROS production nor membrane lipid peroxidation. Although we found, as previously reported, that other common bacteria such as *E. coli* induced sperm ROS production and membrane lipid peroxidation^20^ that was not the case for CT in our experimental setting. The latter results are supported by evidence showing that CT rapidly targets and shuts down NADPH activity thus down-regulating ROS production^38^. However, the exact mechanism of such down-regulation remains to be elucidated.

Our results also indicate that *Chlamydia* spp. infection of the MGT does not alter male fertility. Using a mouse model of male genital infection and performing mating studies with sexually mature female mice, we observed no differences in different fertility parameters, i.e. potency, fecundity, fertility index and embryo loss (before and after implantation), between control and infected males. These data from *in vivo* studies are very valuable since they contemplate the possibility that *Chlamydia* spp. may also damage sperm indirectly through the induction of inflammation or damage of the reproductive organs. Interestingly, our results demonstrate that chlamydia infected male mice showed similar male fertility potential as control males, in agreement with previously reported data in male Wistar rats^22^. Besides, it has been recently reported that CT-infected patients have no increased levels of pro-inflammatory cytokines in semen when compared to control subjects^28^. Moreover, it was reported that once *Chlamydia* spp. infects the genital tract, they induce IL10-secreting B regulatory cells that modulates the induction of protective Th1 immune responses preventing tissue inflammation and favoring the establishment of chronic infections^39^. However, if *Chlamydia* infection ascends to the epididymis or testis, epididymal or testicular function may be affected resulting in an altered sperm quality and compromised male fertility.

The present study also provides evidence suggesting that *Chlamydia* spp. do not attach to spermatozoa *in vitro*. Using electron microscopy, some early studies showed a possible close interaction between CT and sperm cells in biopsy specimens of the testis and epididymis, as well as in semen^17,18,40^. In addition, these studies proposed that CT could be internalized into spermatozoa having important implications for assisted reproduction technology. However, to the best of our knowledge, no similar results have been reported up to date. Moreover, in a later study, these authors failed to confirm that spermatozoa play a role in the pathogenesis of CT infection of the female upper genital tract^41^. Using an animal model of infection in macaques, Patton *et al*. evaluated whether spermatozoa played a role in carrying CT from the infected cervix to the upper female reproductive tract and in the pathogenesis of chlamydial salpingitis. Authors found that, after mating, no spermatozoa examined in samples taken from the upper tract or cul de sac had EBs of CT attached to their surfaces, indicating that spermatozoa do not play a role in the pathogenesis of CT infection. In agreement with Patton *et al*.^41^, results from confocal microscopy and different *in vitro* approaches presented herein also suggest that *Chlamydia* spp. do not attach to spermatozoa. In fact, just washing sperm suspensions previously co-incubated with *Chlamydia* spp. with supplemented BWW medium removed bacteria, since the inoculation of these washed sperm suspensions prevented the further infection of inoculated HeLa cells monolayers or the genital tract of artificially inseminated female mice. Altogether, our results suggest that *Chlamydia* spp. do not attach to spermatozoa *in vitro*. However, additional experimental data are needed to definitely shed light on this issue that has important implications in assisted reproduction technology. In fact, the incidence of CT contamination of sperm samples used in assisted reproduction technology and its impact on their outcomes remain uncertain.

In conclusion, we provide evidence that significantly improves the understanding of sperm cell biology and CT infection, supporting the notion that CT neither directly exerts deleterious effects on spermatozoa nor impairs male fertility. Finally, our results also indicate that CT does not attach to spermatozoa *in vitro* suggesting that these cells would not play a role in the pathogenesis of female genital infection.

## Materials and Methods

### CT strains

Three CT strains were used. In experiments with human sperm, three serovars of CT were used: CT serovar E^21^, CT serovar LGV L2 strain 434/Bu wild type^42^ or transformed with green fluorescent protein (GFP)-expressing plasmid p2TK2-SW2 [GFP-CT]^43^. In experiments with mouse sperm, *C. muridarum* strain Weiss was used^22^. *Chlamydia* spp. EBs were produced and purified as previously described^44^. Briefly, cultures were grown in RPMI-1640 medium supplemented with 20 μg/mL of gentamicin, 5% fetal bovine serum, at 37 °C and 5% CO_2_. Cultures infected with *Chlamydia* spp. were grown for 72 h in the presence of 1 μg/mL of cycloheximide. Infected cell monolayers were detached by scraping and disrupted by sterile glass beads to lyse the host cells and release EBs. Cell debris was removed by centrifugation at 500 × g for 15 min. Chlamydia EBs were purified in a sucrose urografin gradient [bottom layer 50% (w/v) sucrose solution; top layer, 30% (v/v) urografin in 30 mM Tris–HCl buffer, pH 7.4]; at 9000 × *g* and 48 °C for 60 min. After one-wash step with 0.2 mm filtered PBS (pH 7.4), purified EBs were pelleted and resuspended in an isotonic sucrose-phosphate-glutamate (SPG) buffer and aliquots were stored frozen at −80 °C. Infectious titers of this suspension were determined by titration on LLCMK2 cell monolayers. Cultures of infected cells were fixed after 48 h of incubation with methanol 100% and then air dried. Chlamydial inclusions were stained with FITC-monoclonal antibody against chlamydial LPS (Biomerieux, Marcy l′Etoile, France) and analyzed by fluorescence microscopy.

### Animals

A total of 45 male and 80 female C57BL/6 mice aged 6–8 weeks were used. All animals were housed and maintained under specific-pathogen-free (SPF) conditions in the Animal Facility of the Facultad de Ciencias Químicas, Universidad Nacional de Córdoba. Animal were maintained in a 16 h light, 8 h dark cycle, at 20 ± 2 °C, with food and water *ad libitum*.

### Sperm samples

Human semen samples were collected by masturbation after 2–5 days of sexual abstinence from a total of 54 healthy normozoospermic subjects (average age: 30, range: 20–49 years) according to World Health Organization (WHO) criteria^45^. Inclusion criteria were: sperm concentration ≥ 1.5 × 10^7^/mL, total motility ≥ 32%, normal forms ≥ 4%, viability ≥ 58%, leukocyte concentration < 1 × 10^6^/mL and negative sperm and urethral swab cultures (including CT). Fractions of highly motile sperm were isolated using a discontinuous Percoll gradient (45/90%). The final pellet was resuspended in Biggers-Whitten-Wittingham (BWW) medium supplemented with 3% bovine serum albumin (BSA fraction V; Sigma-Aldrich, St. Louis, MO, USA). Murine sperm was obtained from cauda epididymis from male mice. Spermatozoa were incubated in capacitating conditions in BWW medium containing 3% BSA (Sigma-Aldrich) for 30 min at 37 °C in a 5% CO_2_-saturated atmosphere.

Fractions of sperm samples from human and mice individuals were tested separately in a double blind manner for the different assays conducted in the study.

### Ethical approval

This study was conducted between March 2014 and May 2016. Ethical approval for underwent experiments and employed methodologies using human samples was obtained from the Internal Review Board from the Hospital Privado de Córdoba, Córdoba, Argentina (Ref. HP 4–132). The study was conducted in accordance with the Declaration of Helsinki. Each semen donor participated after providing a signed informed consent for his inclusion in the study. All animal experiments were approved and conducted in accordance with guidelines of the Committee for Animal Care and Use of the Facultad de Ciencias Químicas, Universidad Nacional de Córdoba in strict accordance with the recommendation of the Guide for the Care and Use of Laboratory Animals of the NIH (NIH publication 86–23). All efforts were made to minimize suffering and discomfort.

### Sperm incubation with *Chlamydia* spp

Fractions of highly motile human and murine sperm samples were resuspended at a concentration of 1 × 10^6^ sperm/mL and incubated in capacitating conditions (3% BSA supplemented BWW medium) with 1 × 10^5^, 1 × 10^6^ and 1 × 10^7^ EBs/mL of CT (serovar E or LGV) or *C. muridarum*, respectively, at 37 °C during 6 and 24 h (human sperm) or 30 min (murine sperm) following previously described protocols^12–15,46^. Additional sperm fractions were incubated 6 hours in the presence of 1 × 10^6^ colony-forming units (CFU)/mL of uropathogenic *Escherichia coli*, or 0.1 μg/mL chlamydial lipopolysaccharide (cLPS), or 5 μg/mL LPS from *E. coli* (055:B5, Sigma-Aldrich), or soluble products of CT (conditioned media from HeLa cell cultures after 48 h of CT infection), as described^13,14,19^. As controls, fractions of human or murine sperm were incubated with 3% BSA supplemented BWW buffer alone. After incubation, five consecutive washes were performed by adding 500 µL of 3% BSA supplemented BWW buffer to sperm suspensions and then centrifuging at 300 × g for 5 min to remove free bacteria. Finally, different experiments and sperm quality assays were performed. Samples were maintained at 37 °C throughout all procedures.

### Sperm motility and viability

Sperm motility and viability were analyzed according to WHO criteria^45^. Sperm motility was classified into three types: I) progressive motile sperm, II) non-progressive motile sperm and III) immotile sperm. No fewer than 200 gametes were examined. Results are expressed as the percentage of motile cells (progressive plus non-progressive spermatozoa). Sperm viability was evaluated by supravital staining with Eosin Y (Sigma-Aldrich). Spermatozoa that allowed the entry of the dye were classified as dead, and those that excluded the dye were scored as viable. The viability of 200–250 cells was assessed.

### Sperm Apoptosis

Sperm apoptosis was evaluated by Annexin V/PI staining using a commercially available kit (Apoptosis Detection Kit II; BD Pharmingen, San Diego, CA, USA) according to the manufacturer’s instructions, and as previously described^15,46^. Briefly, an aliquot of cells containing 1 × 10^6^ sperms/ml was resuspended in 100 µl binding buffer, labeled with 10 µl Annexin-FITC plus 10 µl PI, incubated for 15 min in the dark, followed by the addition of 400 µl binding buffer and immediately transferred to a flow cytometer and analyzed. The cell population of interest was gated on the basis of its forward and side-scatter properties. The different labeling patterns in the bivariate PI-Annexin V analysis that identified the different cell populations were FITC negative and PI negative and were designated as viable cells; FITC positive and PI negative as early apoptotic cells; FITC positive and PI positive as late apoptotic cells; and FITC negative and PI positive as necrotic cells. A total of 100,000 events were acquired for each sample in a flow cytometer and analyzed. Results are expressed as percentage of cells.

### Sperm ROS production

Sperm intracellular ROS levels were evaluated by flow cytometry using 2′,7′-dichlorodihydrofluorescein diacetate (DCFH-DA, 1 μM; Sigma-Aldrich), a cell-permeable probe highly sensitive to cellular oxidation that fluoresces when oxidized to dichlorodihydrofluorescein (DCFH). PI (Molecular Probes Inc., The Netherlands) was used in conjunction with DCFH-DA as a supravital stain (final concentration: 12 μM). Aliquots of 300 μL containing 2 × 10^6^ spermatozoa were stained with DCFH-DA (final concentration: 1 μM) and incubated for 10 minutes at 37 °C with constant shaking. As positive control, 100 nM of phorbol 12-myristate 13-acetate (PMA) (Sigma-Aldrich) was used. Samples were then analyzed by flow cytometry^46^. Results are expressed as the percentage of fluorescent living cells and the Mean Fluorescence Intensity (MFI).

### Sperm Chromatin Structure Assay

The sperm chromatin structure assay was used to measure the DNA fragmentation index (% DFI). Briefly, at the time of the analysis, aliquots of sperm samples were mixed with 400 μL of a low-pH (pH 1.2) detergent solution containing 0.1% Triton X-100, 0.15 mol/L NaCl, and 0.08 N HCl for 30 sec; this was followed by staining with 1.2 mL 6 mg/mL chromatographically purified acridine orange (AO, Sigma-Aldrich) in phosphate-citrate buffer (pH 6.0). Immediately after the staining, a total of 100,000 events were acquired for each sample in a flow cytometer and analyzed. Under these conditions, AO intercalated into double-stranded DNA emits green fluorescence, and AO associated with single-stranded DNA emits red fluorescence. To avoid instrument drift, reference samples were used to set the red and green photomultiplier tube voltages. In addition, reference negative (vehicle, buffer) and positive (sperm exposed to UV light for 30 min) control samples of sperm were run in every assay. The ratio of single stranded (red) to single plus double stranded (green) fluorescence were expressed as the DFI^47^.

### Mitochondrial membrane potential (ΔΨm)

3,3′-Dihexyloxacarbocyanine iodide [DiOC_6_(3)]/PI was used to detect ΔΨm as previously described^38^. Briefly, a total of 2 × 10^6^ sperms were diluted in 300 μl of 3% BSA supplemented BWW medium. DiOC_6_(3) (Molecular Probes Inc.) was added up to a final concentration of 90 nM. Tubes were gently mixed and incubated for 15 min at room temperature and 5 μl of PI (6 μmol) was added to each tube. Flow cytometry analysis was conducted within 10 min. The test was validated using carbonyl cyanide m‐chlorophenylhydrazone (FCCP), an uncoupler of mitochondrial oxidative phosphorylation, which makes the inner mitochondrial membrane permeable to protons and induces dissipation of ΔΨm. In this control, before adding DiOC_6_(3), 4 μl of 50 mmol/l FCCP were added and the cells were incubated for 30 min at room temperature. Samples were scored as the percentage cells in the population showing high MMP^47^.

### Plasma membrane integrity

The integrity of the spermatozoa plasma membrane was assessed using the LIVE/DEAD® Sperm Viability Kit (Molecular Probes, Inc.). Staining was carried out according to the manufacturer’s instructions. In brief, 300 μl of sperm suspension, containing 2 × 10^6^ sperms, were incubated with SYBR-14 and PI to final concentrations of 1 μM and 12 μM, respectively. Samples were immediately analyzed after by flow cytometry. The data are expressed as percentage of viable sperms positive to SYBR-14 and negative to PI.

### Lipid peroxidation

Oxidative degradation of lipids was measured using the kit BODIPY C11 (Molecular Probes Inc.) according to the method proposed by Aitken *et al*.^48^. In brief, 2 × 10^6^ spermatozoa were incubated with BODIPY C11 (final concentration 5 μM) during 30 min at 25 °C and washed prior to being analyzed by flow cytometry. As positive controls, aliquots of sperm suspensions were additionally treated with t-butyl hydroperoxide (TBHP; 100 mM) to induce lipid peroxidation. The results are expressed as the percentage of cells exhibiting the green fluorescent response.

### Flow cytometry

Flow cytometry analyses were conducted on a FACS CantoII flow cytometer (BD Biosciences), equipped with a 488 nm argon laser. Forward scatter and side scatter measurements were taken to generate a density plot, used to gate for sperm cells. All data were acquired and analyzed using the FlowJo 7.6 software (Tree Star, Ashland, OR, USA) and a total of 10,000–100,000 events were collected per sample and analyzed.

### Chlamydia spp. detection by PCR and qPCR

Detection of chlamydial DNA was performed by the polymerase chain reaction (PCR) in sperm cells fractions pre and post washes and in murine prostate and female genital tract tissue samples as previously described^21–23^. Qualitative and quantitative detection of *C. muridarum* was performed by conventional and real time PCR (qPCR), respectively, by the amplification of the *Chlamydia* spp. *omp2* gene^21–23^. Total DNA was extracted from tissue samples after proteinase K treatment using commercially available extraction columns (AccuPrep® Genomic DNA Extraction Kit, Bioneer, Daejeon, Korea) following the specifications provided by the manufacturer. The primers used for amplification of a 100-bp fragment of the *omp2* gene were: sense 5′-CTG CAA CAG TAT GCG CTT GTC-3′ and antisense 5′-GTC CAC ATT TTG TAA CCG AAC G-3′. The primers used for amplification of a 185 bp fragment of the *eef2* housekeeping gene were as follows: sense 5′-AAG CTG ATC GAG AAG CTG GA -3′ and antisense 5′-CCC CTC GTA TAG CAG CTC AC -3′. Conventional PCR was performed as previously described^23^. A sample was considered positive when amplification of the *omp2* gene was detected. Quantitative real time PCR was performed on a StepOneTM instrument (Life technologies, Carlsbad, CA, USA) using SYBR® Select Master Mix (Life technologies). Each qPCR experiment was performed at least in triplicates, in a final volume of 15 µL. Each reaction contained 2 µL of DNA template and 1 µM of each primer (sense/antisense) of both genes (*omp2* target gene and *eef2* housekeeping gene). A single initial denaturation step of 15 min at 95 °C was followed by 40 cycles of 15 sec at 95 °C (denaturation), 30 sec at 60 °C (annealing) and 30 sec at 72 °C (elongation). Negative and positive controls were included in each run. After performing thermal cycling, qPCR amplification data were analyzed using StepOne software (Applied Biosystems, Foster City, CA, USA).

### Assessment of CT attachment to human spermatozoa by confocal microscopy

Fractions of highly motile human sperm samples containing 1 × 10^6^ spermatozoa/mL were *in vitro* incubated with 3% BSA supplemented BWW medium alone or with 1 × 10^5^, 1 × 10^6^ and 1 × 10^7^ EBs/mL of green fluorescent GFP-CT during 6 h at 37 °C, as described above. After five consecutive washes and centrifuging at 300 x*g* for 5 min to remove free bacterial EBs, sperm smears were prepared on glass slides, air-dried, fixed with 4% paraformaldehyde in PBS pH 7.4 at room temperature for 10 min, stained with DAPI (Wako Pure Chemical Industries, Richmond, VA, USA) and counterstained with 0.01% Evans blue for 10 minutes. Slides mounted with FluorSave (Calbiochem, San Diego, CA, USA) were visualized with an Olympus FV1200 laser scanning confocal microscope (Olympus, Center Valley, PA, USA). Images were captured using an objective PLAPON 60X (1.42 NA). Fluorophore signals were acquired in sequential mode and images were analyzed using Olympus software (Olympus).

### Infection assessment of *in vitro* cultured HeLa cells after inoculation with sperm pre-incubated with *Chlamydia* spp

Seventy to 80% confluent HeLa cell monolayers cultured at 37 °C/5% CO_2_ were inoculated with 1 × 10^6^ human or murine spermatozoa pre-incubated *in vitro* with 1 × 10^5^, 1 × 10^6^ or 1 × 10^7^ EBs of CT or *C. muridarum*/million sperm, respectively, before and after 5 consecutive washes as described above. After 48 h of culture, infection of cultures was assessed by the detection of chlamydial inclusion bodies, as previously described^44^. Cultures were fixed after 48 h of incubation with methanol 100% and then air dried. To detect chlamydial inclusion bodies, cell monolayers were then stained with FITC-monoclonal antibody against chlamydial LPS (Biomerieux) and analyzed by fluorescence microscopy.

### Determination of chlamydial infection in the genital tract of female mice inseminated with murine sperm *in vitro* pre-incubated with *C. muridarum*

To test whether *C. muridarum* attaches to murine spermatozoa, experiments using a mouse model of infection^49^ were also performed. Six-8 week old female C57BL/6 mice received three subcutaneous doses of 2.5 mg/mouse of medroxyprogesterone acetate (Sigma-Aldrich) on days 13, 10 and 3 before vaginal insemination. To confirm that all mice were on diestrous, vaginal cytology was checked before insemination. Murine sperm were *in vitro* incubated with EBs of *C. muridarum* in capacitating conditions, washed 5 times and then intravaginally inoculated in *oestrous* female C57BL/6 mice, as described above. Four experimental groups were included: the sham infected group inseminated with 30 µL of a solution containing 1 × 10^6^ sperm pre-incubated with vehicle alone (BWW buffer, negative control group); the positive control group inseminated with 30 µL of a solution containing 1 × 10^6^ sperm pre-incubated with 1 × 10^7^ EBs of *C. muridarum* without subsequent washings; one group inseminated with 30 µL of a solution containing 1 × 10^6^ sperm pre-incubated with 1 × 10^6^ EBs of *C. muridarum* and then washed 5 times; and one group inseminated with 30 µL of a solution containing 1 × 10^6^ sperm pre-incubated with 1 × 10^7^ EBs of *C. muridarum* and then washed 5 times. To assay infection, vaginal lavages were collected on day 7 after insemination; then, animals were euthanized on day 15 after insemination and vagina, uterus and ovaries were excised. Total DNA extraction from vaginal swabs and tissue samples was performed and the presence of *C. muridarum* was assayed by PCR and qPCR as described above.

### Animal model of *C. muridarum* infection of the MGT

As previously described^23^, 6–8 week old male C57BL/6 mice were inoculated in the meatus urethra with 1 × 10^8^ EBs of *C. muridarum* in 20 μL of SPG. For the inoculation, animals (n = 6) were anesthetized and put on their backs, the prepuce was pulled back and the inoculum was placed on the meatus urethra, simulating its natural infection path to the genital tract. A control group of animals (n = 6) was sham infected with 20 μL of SPG. This time point was considered day 0 of infection. After 15 days of infection, males were mated with sexually mature female C57BL/6 mice in order to assess different male fertility parameters. Then, males from the infected and control groups were euthanized (20 days post inoculation) to confirm *C. muridarum* infection of the MGT. Once animals were euthanized, the prostate was excised. Total DNA extraction from prostate tissue samples was performed and the presence of *C. muridarum* was assayed by PCR and qPCR as described above.

### Mating Studies

Sham infected and infected male C57BL/6 mice were mated with normally cycling female C57BL/6 mice, as previously described^22^. Six males per experimental group were mated to 3 females each. A total of 36 females were tested, including 18 females per infected group and 18 per sham infected/control group. On the morning of proestrus-estrous each female was transferred to the appropriate male cage at approximately 5:00 p.m. for overnight breeding. Females were removed from the male cages early the next morning. Sperm or a copulatory plug in the vaginal lavage was considered evidence of mating. This day was designated day 0 of gestation.

### Fertility Parameters

Females were sacrificed on day 18 of gestation. The uterus was exposed and the number of implantation sites was recorded. Ovaries were exposed to count the number of corpora lutea. Live, dead and resorbed fetuses were noted. Potency was calculated as the ratio of females inseminated to the number exposed for mating × 100. The fertility index was calculated as the ratio of the number of implantation sites to the number of corpora lutea. Fecundity was calculated as the ratio of the number of males that sired at least 1 viable fetus to the total number exposed for mating × 100. Pre-implantation embryo loss was determined by the equation, (number of corpora lutea - number of implantation sites/number of corpora lutea) × 100. Post-implantation embryo loss was determined by the equation, (number of implantation sites - number of live fetuses/number of implantation sites) × 100.

### Statistical analyses

Statistical analysis was performed using one-way or two-way ANOVA with Bonferroni post hoc test analysis. Mean ± SD are represented in the graphs. Statistical tests were performed using the GraphPad Prism 5.0 software. The *p* value < 0.05 was considered significant in all analyses. Compromise power analyses using G*Power3 data analysis program^50^ was performed to determine the statistical power (>0.86) to exclude the possibility of type II error.

## Electronic supplementary material

Supplementay

## References

[1] Brunham RC, Rappuoli R (2013). Chlamydia trachomatis control requires a vaccine. Vaccine.

[2] Senior K (2012). Chlamydia: a much underestimated STI. Lancet Infect. Dis..

[3] Dimech W (2014). ACCESS collaboration. Analysis of laboratory testing results collected in an enhanced chlamydia surveillance system in Australia, 2008–2010. BMC Infect. Dis..

[4] Paavonen J (2012). Chlamydia trachomatis infections of the female genital tract: state of the art. Ann. Med..

[5] Wagenlehner FM, Weidner W, Pilatz A, Naber KG (2014). Urinary tract infections and bacterial prostatitis in men. Curr. Opin. Infect. Dis..

[6] Mackern-Oberti JP (2013). Chlamydia trachomatis infection of the male genital tract: an update. J. Reprod. Immunol..

[7] Redgrove KA, McLaughlin EA (2014). The Role of the Immune Response in Chlamydia trachomatis Infection of the Male Genital Tract: A Double-Edged Sword. Front. Immunol.

[8] Gimenes F (2014). Male infertility: a public health issue caused by sexually transmitted pathogens. Nature Rev. Urol.

[9] Cunningham KA, Beagley KW (2008). Male genital tract chlamydial infection: implications for pathology and infertility. Biol. Reprod.

[10] Joki-Korpela P (2009). The role of Chlamydia trachomatis infection in male infertility. Fertil. Steril..

[11] Hosseinzadeh S, Brewis IA, Pacey AA, Moore HD, Eley A (2000). Coincubation of human spermatozoa with Chlamydia trachomatis *in vitro* causes increased tyrosine phosphorylation of sperm proteins. Infect. Immun..

[12] Hosseinzadeh S, Brewis IA, Eley A, Pacey AA (2001). Co-incubation of human spermatozoa with Chlamydia trachomatis serovar E causes premature sperm death. Hum. Reprod.

[13] Hosseinzadeh S, Pacey AA, Eley A (2003). Chlamydia trachomatis-induced death of human spermatozoa is caused primarily by lipopolysaccharide. J. Med. Microbiol.

[14] Eley A, Hosseinzadeh S, Hakimi H, Geary I, Pacey AA (2005). Apoptosis of ejaculated human sperm is induced by co-incubation with Chlamydia trachomatis lipopolysaccharide. Hum. Reprod..

[15] Satta A (2006). Experimental Chlamydia trachomatis infection causes apoptosis in human sperm. Hum. Reprod..

[16] Hosseinzadeh S, Eley A, Pacey AA (2004). Semen quality of men with asymptomatic chlamydial infection. J. Androl.

[17] Erbengi T (1993). Ultrastructural observations on the entry of Chlamydia trachomatis into human spermatozoa. Hum. Reprod.

[18] Villegas H, Pinon M, Shor V, Karchmer S (1991). Electron microscopy of Chlamydia trachomatis infection of the male genital tract. Arch. Androl..

[19] Schulz M, Sanchez R, Soto L, Risopatron J, Villegas J (2010). Effect of Escherichia coli and its soluble factors on mitochondrial membrane potential, phosphatidylserine translocation, viability, and motility of human spermatozoa. Fertil. Steril..

[20] Barbonetti A (2013). Soluble products of Escherichia coli induce mitochondrial dysfunction-related sperm membrane lipid peroxidation which is prevented by lactobacilli. PLoS ONE.

[21] Mackern-Oberti JP (2011). Male rodent genital tract infection with Chlamydia muridarum: persistence in the prostate gland that triggers self-immune reactions in genetically susceptible hosts. J. Urol..

[22] Motrich RD, Sanchez L, Maccioni M, Mackern-Oberti JP, Rivero VE (2012). Male rat genital tract infection with Chlamydia muridarum has no significant consequence on male fertility. J. Urol..

[23] Sanchez LR (2017). Chronic infection of the prostate by Chlamydia muridarum is accompanied by local inflammation and pelvic pain development. The Prostate.

[24] Bachir BG, Jarvi K (2014). Infectious, inflammatory, and immunologic conditions resulting in male infertility. Urol. Clin. North. Am..

[25] Ljubin-Sternak S, Mestrovic T (2014). Chlamydia trachomatis and Genital Mycoplasmas: Pathogens with an Impact on Human Reproductive Health. J. Pathog.

[26] Mazzoli S (2010). Chlamydia trachomatis infection is related to poor semen quality in young prostatitis patients. Eur. Urol..

[27] Pajovic B, Radojevic N, Vukovic M, Stjepcevic A (2013). Semen analysis before and after antibiotic treatment of asymptomatic Chlamydia- and Ureaplasma-related pyospermia. Andrologia.

[28] Dehghan Marvast, L., Aflatoonian, A., Talebi, A.R., Ghasemzadeh, J. & Pacey, A.A. Semen inflammatory markers and Chlamydia trachomatis infection in male partners of infertile couples. *Andrologia* (2016). In press. 10.1111/and.12501 26646684

[29] Eggert-Kruse W, Batschulat K, Demirakca T, Strowitzki T (2015). Male immunity to the chlamydial 60 kDa heat shock protein (HSP 60)-associated with semen quality?. Andrologia.

[30] Vigil P, Morales P, Tapia A, Riquelme R, Salgado AM (2002). Chlamydia trachomatis infection in male partners of infertile couples: incidence and sperm function. Andrologia.

[31] Eggert-Kruse W (2003). Prevalence of Chlamydia trachomatis in subfertile couples. Fertil. Steril..

[32] Motrich RD, Cuffini C, Oberti JP, Maccioni M, Rivero VE (2006). Chlamydia trachomatis occurrence and its impact on sperm quality in chronic prostatitis patients. J. Infect..

[33] Weidner W, Floren E, Zimmermann O, Thiele D, Ludwig M (1996). Chlamydial antibodies in semen: search for “silent” chlamydial infections in asymptomatic andrological patients. Infection.

[34] Gallegos G (2008). Sperm DNA fragmentation in infertile men with genitourinary infection by Chlamydia trachomatis and Mycoplasma. Fertil. Steril..

[35] Cai T (2014). Effect of human papillomavirus and Chlamydia trachomatis co-infection on sperm quality in young heterosexual men with chronic prostatitis-related symptoms. BJU Int..

[36] Sobinoff AP (2015). Chlamydia muridarum infection-induced destruction of male germ cells and sertoli cells is partially prevented by Chlamydia major outer membrane protein-specific immune CD4 cells. Biol. Reprod.

[37] Dehghan Marvast, L., Aflatoonian, A., Talebi, A.R., Eley, A. & Pacey, A.A. Relationship between Chlamydia trachomatis and Mycoplasma genitalium infection and pregnancy rate and outcome in Iranian infertile couples. *Andrologia*. (2016). In press. 10.1111/and.12747 28032361

[38] Boncompain G (2010). Production of reactive oxygen species is turned on and rapidly shut down in epithelial cells infected with Chlamydia trachomatis. Infect. Immun..

[39] Moore-Connors JM (2015). CD43-, but not CD43+, IL-10-producing CD1dhiCD5+B cells suppress type 1 immune responses during Chlamydia muridarum genital tract infection. Mucosal Immunol.

[40] Wolner-Hanssen P, Mardh PA (1984). *In vitro* tests of the adherence of Chlamydia trachomatis to human spermatozoa. Fertil. Steril..

[41] Patton DL (1993). The role of spermatozoa in the pathogenesis of Chlamydia trachomatis salpingitis in a primate model. Sex. Transm. Dis..

[42] Saka HA (2015). Chlamydia trachomatis Infection Leads to Defined Alterations to the Lipid Droplet Proteome in Epithelial Cells. PLoS ONE.

[43] Agaisse H, Derré I (2013). A C. trachomatis cloning vector and the generation of C. trachomatis strains expressing fluorescent proteins under the control of a C. trachomatis promoter. PLoS One.

[44] Mackern-Oberti JP, Maccioni M, Cuffini C, Gatti G, Rivero VE (2006). Susceptibility of prostate epithelial cells to Chlamydia muridarum infection and their role in innate immunity by recruitment of intracellular Toll-like receptors 4 and 2 and MyD88 to the inclusion. Infect. Immun..

[45] World Health Organization. WHO Laboratory Manual for the Examination and Processing of Human Semen (5th Ed.) (WHO 2010).

[46] Motrich RD (2006). Pathogenic consequences in semen quality of an autoimmune response against the prostate gland: from animal models to human disease. J. Immunol..

[47] Mayorga-Torres BJ (2015). Influence of ejaculation frequency on seminal parameters. Reprod. Biol. Endocrinol..

[48] Aitken RJ, Wingate JK, De Iuliis GN, McLaughlin EA (2007). Analysis of lipid peroxidation in human spermatozoa using BODIPY C11. Mol. Hum. Reprod..

[49] Slepenkin A, Chu H, Elofsson M, Keyser P, Peterson EM (2011). Protection of mice from a Chlamydia trachomatis vaginal infection using a Salicylidene acylhydrazide, a potential microbicide. J. Infect. Dis..

[50] Faul F, Erdfelder E, Lang AG, Buchner A (2007). G*Power 3: A flexible statistical power analysis program for the social, behavioral, andbiomedical sciences. Behav. Res. Methods.

