# Erratum: *Chlamydia trachomatis* neither exerts deleterious effects on spermatozoa nor impairs male fertility

**DOI:** 10.1038/s41598-017-12316-4

**Published:** 2017-11-21

**Authors:** Jenniffer Puerta Suárez, Leonardo R. Sánchez, Florencia C. Salazar, Hector A. Saka, Rosa Molina, Andrea Tissera, Virginia E. Rivero, Walter D. Cardona Maya, Ruben D. Motrich

**Affiliations:** 1 Centro de Investigaciones en Bioquímica Clínica e Inmunología (CIBICI-CONICET), Departamento de Bioquímica Clínica. Facultad de Ciencias Químicas, Universidad Nacional de Córdoba, Haya de la Torre y Medina Allende, Córdoba, 5016 Argentina; 2 Grupo Reproducción, Facultad de Medicina, Universidad de Antioquia, Sede de Investigación Universitaria, SIU, Laboratorio 534, 1226 Medellín, Colombia; 3 Laboratorio de Andrología y Reproducción, LAR, Córdoba 5016 Argentina

*Scientific Reports* **7**:1126; doi:10.1038/s41598-017-01262-w; Article published online 25 April 2017

The Acknowledgements section in this Article is incomplete:

“We thank Paula Icely, Paula Abadie, Pilar Crespo, Carlos Mas and Jose Manuel Mayorga Torres for excellent technical assistance. We also thank Carolina Florit, Victoria Blanco, Fabricio Navarro and Diego Lutti for animal care.”

should read:

“We thank Paula Icely, Paula Abadie, Pilar Crespo, Carlos Mas and Jose Manuel Mayorga Torres for excellent technical assistance. We also thank Carolina Florit, Victoria Blanco, Fabricio Navarro and Diego Lutti for animal care.

This study was supported by the Secretaría de Ciencia y Tecnología de la Universidad Nacional de Córdoba (grants Cat. A and B, SECyT-UNC), CONICET (special grants for young researchers), Agencia Nacional de Promoción Científica y Tecnológica (grants PICT 2008-1639, PICT 2012-1417, PICT 2013-2201 and PICT 2014-2195), and COLCIENCIAS, Colombia (grant#111556933373).

J.P.S. was a fellow from the Programa Latinoamericano de Investigación en Salud Sexual y Reproductiva [PLISSER]. R.D.M. and V.E.R. are members of the Scientific Career of CONICET.”

