# Supplementary material for: Chlamydia trachomatis neither exerts deleterious effects on spermatozoa nor impairs male fertility
Source: Sci Rep. 2017 Apr 25;7:1126. doi: 10.1038/s41598-017-01262-w (PMC5430866; doi:10.1038/s41598-017-01262-w)
Supplement: Supplementary file 1 — Supplementay [file 41598_2017_1262_MOESM1_ESM.pdf]

Supplementary information

Chlamydia trachomatis neither exerts deleterious effects on spermatozoa nor impairs male fertility.

Jenniffer Puerta-Suárez, Leonardo R. Sánchez, Florencia C. Salazar, Hector A. Saka, Rosa Molina,Andrea Tissera, Virginia E. Rivero, Walter D. Cardona-Maya, Ruben D. Motrich.

Supplementary Table 1: *Effects of soluble factors from CT on the mitochondrial physiology and DNA integrity of human sperm*.

**Sperm mitochondrial membrane potential, ΔΨm (%) [a]**								
Condition	Controls		Soluble factors from CT serovar E			Soluble factors from CT serovar LGV		
Sample	Vehicle	Control +	$1x10^5$ EBs	$1x10^6$ EBs	$1x10^7$ EBs	$1x10^5$ EBs	$1x10^6$ EBs	$1x10^7$ EBs
High ΔΨm	41.4 ± 8.9	30.1 ± 4.8	44.8 ± 8.4	46.1 ± 8.7	46.3 ± 7.8	46.0 ± 7.7	46.3 ± 8.1	46.3 ± 8.5
Low ΔΨm	6.8 ± 2.4	3.1 ± 1.4	6.3 ± 3.3	7.4 ± 3.8	6.5 ± 2.2	5.7 ± 2.3	7.7 ± 3.8	7.0 ± 2.7

**Sperm DNA fragmentation [b]**								
Condition	Controls		Soluble factors from CT serovar E			Soluble factors from CT serovar LGV		
Sample	Vehicle	Control +	$1x10^5$ EBs	$1x10^6$ EBs	$1x10^7$ EBs	$1x10^5$ EBs	$1x10^6$ EBs	$1x10^7$ EBs
DNA fragmentation index	9.0 ± 4.9	18.9 ± 3.6	11.1 ± 5.1	13.5 ± 7.8	11.6 ± 5.1	11.5 ± 4.3	11.3 ± 4.2	11.0 ± 5.2

Percentages of human sperm exhibiting high and low mitochondrial membrane potential (ΔΨm) [a], and evaluation of DNA fragmentation [b], in human sperm samples after 6 h of *in vitro* incubation with soluble factors (conditioned media) from HeLa cell cultures infected with increasing concentrations of EBs of CT serovar E, or serovar LGV, with control DMEM medium (DMEM), or with BWW medium (Vehicle). Data are shown as mean ± SD, n=36. Statistical analysis was performed using one-way ANOVA with Bonferroni post hoc test analysis ($p>0.05$).

Supplementary Figure 1

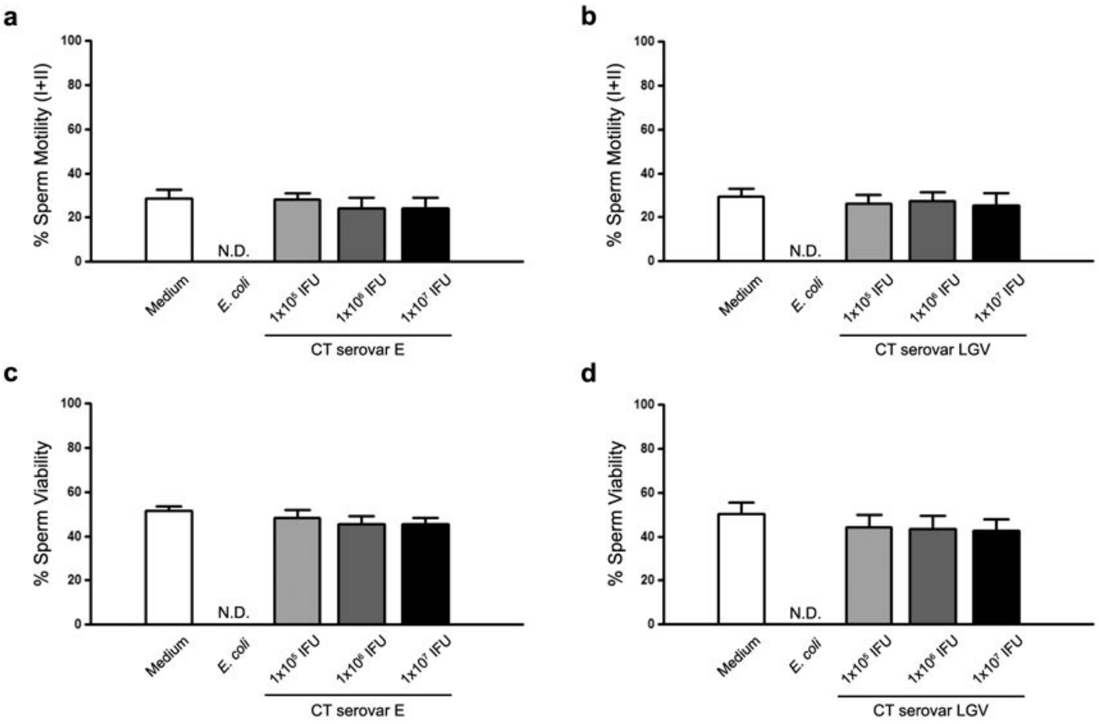

Supplementary Figure 2

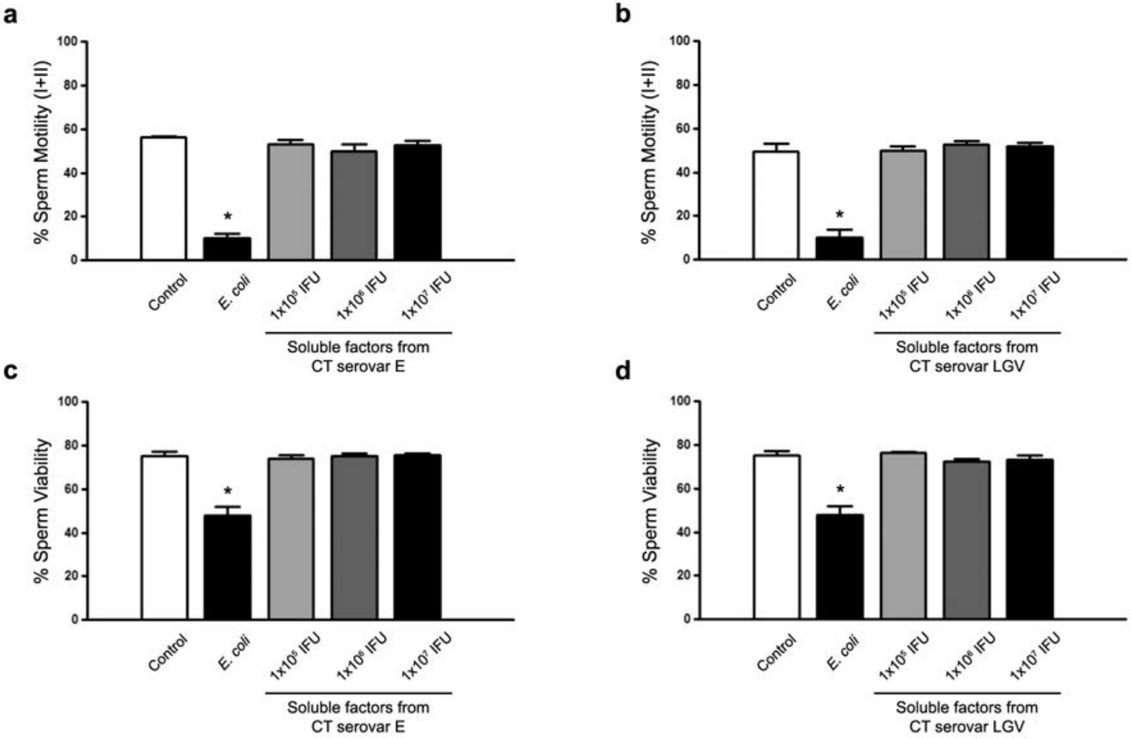

e

Sperm membrane integrity (%)								
	Controls		Soluble factors from CT serovar E			Soluble factors from CT serovar LGV		
Sample	Medium	*E. coli*	$1x10^5$ EBs	$1x10^6$ EBs	$1x10^7$ EBs	$1x10^5$ EBs	$1x10^6$ EBs	$1x10^7$ EBs
Live cells	77,0 ± 14,6	29,4 ± 7,8 *	76,6 ± 13,5	78,7 ± 14,8	77,1 ± 13,3	75,1 ± 12,3	72,1 ± 21,1	70,9 ± 18,9
Dying cells	2,6 ± 1,7	17,3 ± 13,1 *	4,9 ± 6,0	2,9 ± 2,4	3,4 ± 3,2	6,7 ± 5,1	7,9 ± 8,3	7,6 ± 9,9
Necrotic cells	20,4 ± 14,5	53,3 ± 9,6 *	18,5 ± 12,8	18,4 ± 13,0	19,5 ± 15,5	18,2 ± 4,8	20,0 ± 6,9	21,5 ± 13,6

Supplementary Figure 3

a

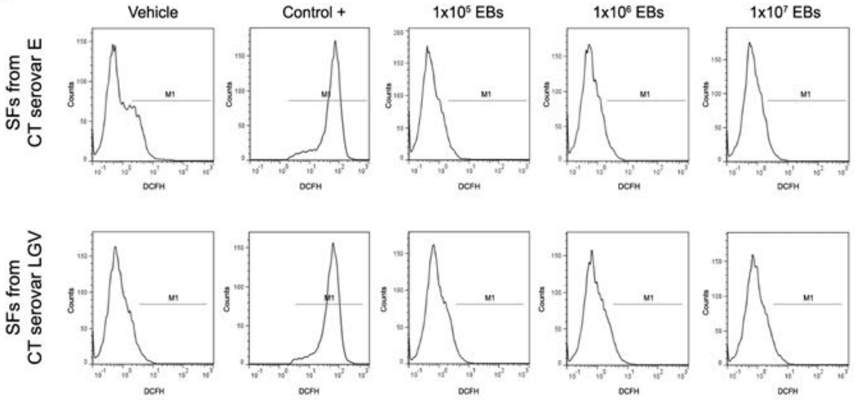

b

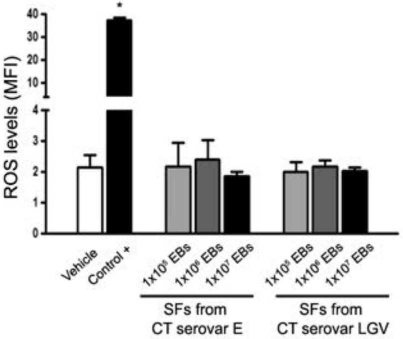

c

Sperm membrane lipid peroxidation								
Condition	Controls		SFs from CT serovar E			SFs from CT serovar LGV		
Sample	Vehicle	Control +	1x10^5 EBs	1x10^6 EBs	1x10^7 EBs	1x10^5 EBs	1x10^6 EBs	1x10^7 EBs
Green fluorescent BODIPY C11 cells (%)	6,8 ± 1,6	90,1 ± 4,8 *	6,8 ± 1,7	6,8 ± 1,6	7,0 ± 1,8	6,6 ± 1,5	7,1 ± 0,9	7,0 ± 1,1

Supplementary Figure Legends

Supplementary Figure 1: *Effects of Chlamydia spp. on sperm motility and viability.* Human sperm motility (%) after 24 h of *in vitro* incubation without bacteria (Control), or with increasing concentrations of EBs of CT serovar E (**a**) or serovar LGV (**b**) per million spermatozoa. Human sperm viability (%) after 24 h of *in vitro* incubation without bacteria (Control), or with increasing concentrations of EBs of CT serovar E (**c**) or serovar LGV (**d**) per million spermatozoa. As positive controls, sperm fractions were incubated with uropathogenic *E. coli* ($1x10^6$ CFU/mL). Data are shown as mean ± SD. Fractions of human (n=15) sperm samples were tested separately and maintained at 37°C throughout all procedures. Statistical analysis was performed using one-way ANOVA with Bonferroni post hoc test analysis and no significant differences were found in any condition ($p<0.05$).

Supplementary Figure 2: *Effects of soluble factors from CT on human sperm motility and viability.* Human sperm motility (%) after 6 h of *in vitro* incubation with soluble factors (conditioned media) from HeLa cells cultures after the infection with increasing concentrations of EBs of CT serovar E (**a**), or serovar LGV (**b**), with control DMEM medium (Control), or with uropathogenic *E. coli* ($1x10^6$ CFU/mL) as positive control. Human sperm viability (%) after 6 h of *in vitro* incubation with soluble factors (conditioned media) from HeLa cells cultures infected with increasing concentrations of EBs of CT serovar E (**c**), or serovar LGV (**d**), with control DMEM medium (Control), or with uropathogenic *E. coli* ($1x10^6$ CFU/mL) as positive control. (**e**) Sperm membrane integrity (%) in human spermatozoa after *in vitro* incubation with soluble factors

(conditioned media) from HeLa cells cultures infected with increasing concentrations of EBs of CT serovar E, or serovar LGV, with control DMEM medium (Medium), or with uropathogenic *E. coli* ($1x10^6$ CFU/mL) as positive control. Data are shown as mean ± SD. Fractions of human samples (n=36) were tested separately and maintained at 37°C throughout all procedures. Statistical analysis was performed using one-way ANOVA with Bonferroni post hoc test analysis ($p<0.05$).

Supplementary Figure 3: *ROS production and lipid peroxidation in human spermatozoa exposed to soluble factors from CT*. Analysis of ROS production by human spermatozoa after 6 h of *in vitro* incubation with soluble factors (conditioned media) from HeLa cells cultures infected with increasing concentrations of EBs of CT serovar E, or serovar LGV, with control DMEM medium (Vehicle), or with 100 nM PMA (positive control). ROS production was assessed by flow cytometry using the probe DCFH-DA that fluoresces when oxidized to DCFH. (**a**) Histograms show sperm with ROS production (M1). (**b**) ROS production levels (MFI) are shown in bars. (**c**) Human sperm membrane lipid peroxidation (%) after 6 h of *in vitro* incubation with soluble factors (conditioned media) from HeLa cell cultures infected with increasing concentrations of EBs of CT serovar E, or serovar LGV, with control DMEM medium (Vehicle), or with 100 mM TBHP (positive control). Lipid peroxidation was analyzed using BODIPY C11. Data are shown as mean ± SD. Fractions of sperm samples (n=36) were tested separately. Statistical analysis was performed using one-way ANOVA with Bonferroni post hoc test analysis ($p<0.05$).
